# The involvement of cation leaks in the storage lesion of red blood cells

**DOI:** 10.3389/fphys.2014.00214

**Published:** 2014-06-17

**Authors:** Joanna F. Flatt, Waleed M. Bawazir, Lesley J. Bruce

**Affiliations:** ^1^Bristol Institute for Transfusion Sciences, NHS Blood and TransplantBristol, UK; ^2^School of Biochemistry, University of BristolBristol, UK

**Keywords:** red blood cell storage, storage lesion, cation leak, vesiculation, oxidation, transfusion

## Abstract

Stored blood components are a critical life-saving tool provided to patients by health services worldwide. Red cells may be stored for up to 42 days, allowing for efficient blood bank inventory management, but with prolonged storage comes an unwanted side-effect known as the “storage lesion”, which has been implicated in poorer patient outcomes. This lesion is comprised of a number of processes that are inter-dependent. Metabolic changes include a reduction in glycolysis and ATP production after the first week of storage. This leads to an accumulation of lactate and drop in pH. Longer term damage may be done by the consequent reduction in anti-oxidant enzymes, which contributes to protein and lipid oxidation via reactive oxygen species. The oxidative damage to the cytoskeleton and membrane is involved in increased vesiculation and loss of cation gradients across the membrane. The irreversible damage caused by extensive membrane loss via vesiculation alongside dehydration is likely to result in immediate splenic sequestration of these dense, spherocytic cells. Although often overlooked in the literature, the loss of the cation gradient in stored cells will be considered in more depth in this review as well as the possible effects it may have on other elements of the storage lesion. It has now become clear that blood donors can exhibit quite large variations in the properties of their red cells, including microvesicle production and the rate of cation leak. The implications for the quality of stored red cells from such donors is discussed.

## Introduction

Stored red blood cells (RBCs) have provided life-saving transfusions for many years. Over this time improvements in storage techniques and media have increased the viability and functionality of stored RBCs. The development of storage solutions (reviewed in Moore, 1987; Hess, 2006) citrate-phosphate-dextrose (CPD) and acid-citrate-dextrose (ACD) (Weisert and Jeremic, 1973), saline-adenine-glucose (SAG) (Ambrus et al., 1975; Kreuger et al., 1975; Herve et al., 1980; Strauss et al., 1980; Peck et al., 1981), with added mannitol (Högman et al., 1983) as in the SAG-M additive commonly used in the UK today, or the more recent development of chloride-free additives (Högman et al., 2006) and phosphate-adenine-glucose-guanosine-gluconate-mannitol (PAGGGM) (Burger et al., 2010), has helped to preserve metabolic functions and reduce lipid peroxidation (Knight et al., 1993). Leukoreduction, which was introduced in the UK in the late 1990s (AuBuchon et al., 1997; reviewed in Roddie et al., 2000) primarily to reduce the transmission of viruses, was shown to decrease hemolysis (Williamson et al., 1999) and the oxidative damage and calcium-related stress of stored RBCs (Antonelou et al., 2012). Nonetheless, stored RBCs still deteriorate during storage in ways that are not fully understood (Hess, 2012) and this “storage lesion” has been implicated in the poor outcome, post-transfusion, of certain categories of patients (Wang et al., 2012).

In the UK RBCs may be stored for 35 days prior to transfusion. During this time the “storage lesion” develops, characterized by changes in cation gradients, metabolism, oxidation, and vesiculation. Although there has been a lot of research into the different elements of the storage lesion, it is not yet clear in what order these defects occur and the sequence of cause and effect (Hess, 2010). Furthermore, very little attention has been given to the cation leak and how it contributes to, or maybe even triggers, other elements of the storage lesion. In this paper we will review the literature on RBC storage. We will consider the time-line of storage, the cause and effect of the different elements of the storage lesion, and we will describe certain variations that occur in the properties of donor blood. Other aspects of RBC storage are discussed in two companion papers of this research topic “Regulation of red cell life-span, erythropoiesis, senescence and clearance” (Bosman, 2013; Lutz and Bogdanova, 2013).

### Donor RBCs are a mixed population

Donor RBCs are a mixed population of cells at the time of donation, ranging from newly formed reticulocytes through to 120 day old RBCs that are about to be removed from circulation. There is some evidence to suggest that during storage RBCs are in a state of suspended animation and do not continue to “age” as they would in the circulation at body temperature. The protein 4.1a/b ratio, an indicator of RBC age, doesn't change through storage (Minetti et al., 2001). A recent study on reticulocyte maturation, by our group in Bristol, also provided evidence that reticulocytes do not mature in storage. In this study it was shown that reticulocyte maturation involves the formation of endocytic vesicles which then merge with autophagic vesicles forming large GPA and LC3 (autophagy marker) positive vesicles containing mitochondria etc. (Griffiths et al., 2012). These large internal GPA/LC3 positive vesicles can be seen in a small number of RBCs throughout the 35 days storage period suggesting that reticulocytes in donor blood at the time of donation do not mature significantly during the storage period. So at any given moment during RBC storage the unit will still contain a mixed population of cells from reticulocytes through to pre-senescent RBCs. Whether these RBCs, at different stages of maturity, succumb to the storage lesion equally has not been established but there are some indications that the lesion may affect young, middle and aged stored RBCs differently (Snyder et al., 1985).

### Metabolic changes

The metabolic changes that occur in RBCs during storage have been extensively studied (Brewer et al., 1976; Strauss et al., 1980; Messana et al., 2000; reviewed in Hess and Greenwalt, 2002; Kanias and Acker, 2010; Buehler et al., 2011; Hess, 2014). RBCs lack mitochondria and are completely dependent on glycolysis for their energy requirements. RBCs are stored at 4°C, a temperature that slows metabolism, reducing the production of ATP and any RBC functions that require energy. The rate of glycolysis depends on temperature but more importantly on pH. The pH also affects the 2,3-diphosphoglycerate (2,3-DPG) level which in turn affects the oxygen carrying capacity of hemoglobin (Hb). Consequently, preservation of 2,3-DPG and adenosine triphosphate (ATP) levels by improved RBC storage solutions has been the focus of much research (Ambrus et al., 1975; Peck et al., 1981; Högman et al., 2006; reviewed in Hess and Greenwalt, 2002). A recent study reported in detail on the time-course of changes in metabolites during RBC storage (D'Alessandro et al., 2012). In summary their findings show that concentrations of metabolites such as fructose 1,6-diphosphate, glyceraldehyde-3-phosphate, total diphosphoglycerate, nicotinamide adenine dinucleotide (NAD^+^) and ATP, all involved in the glycolytic pathway, increase during the first 7 days of storage, suggesting that at this early stage of storage glycolysis is proceeding. This increase in metabolites during the first 7 days of storage may in part be caused by the formation of deoxyhemoglobin which binds competitively to the N-terminal domain of band 3 (SLC4A1), displacing glycolytic enzymes and thereby activating them (Low et al., 1993; Messana et al., 2000). However, after day 7 the concentrations of these metabolites fall and that of phosphoenolpyruvate increases suggesting that glycolysis slows down. This is probably caused by the drop in pH, as lactic acid builds up, inhibiting glycolysis via a negative feedback mechanism. Lactate is excreted by the RBC *in vivo* and processed by the liver, however during blood storage lactate inevitably builds up in the bag.

**Figure 1:**
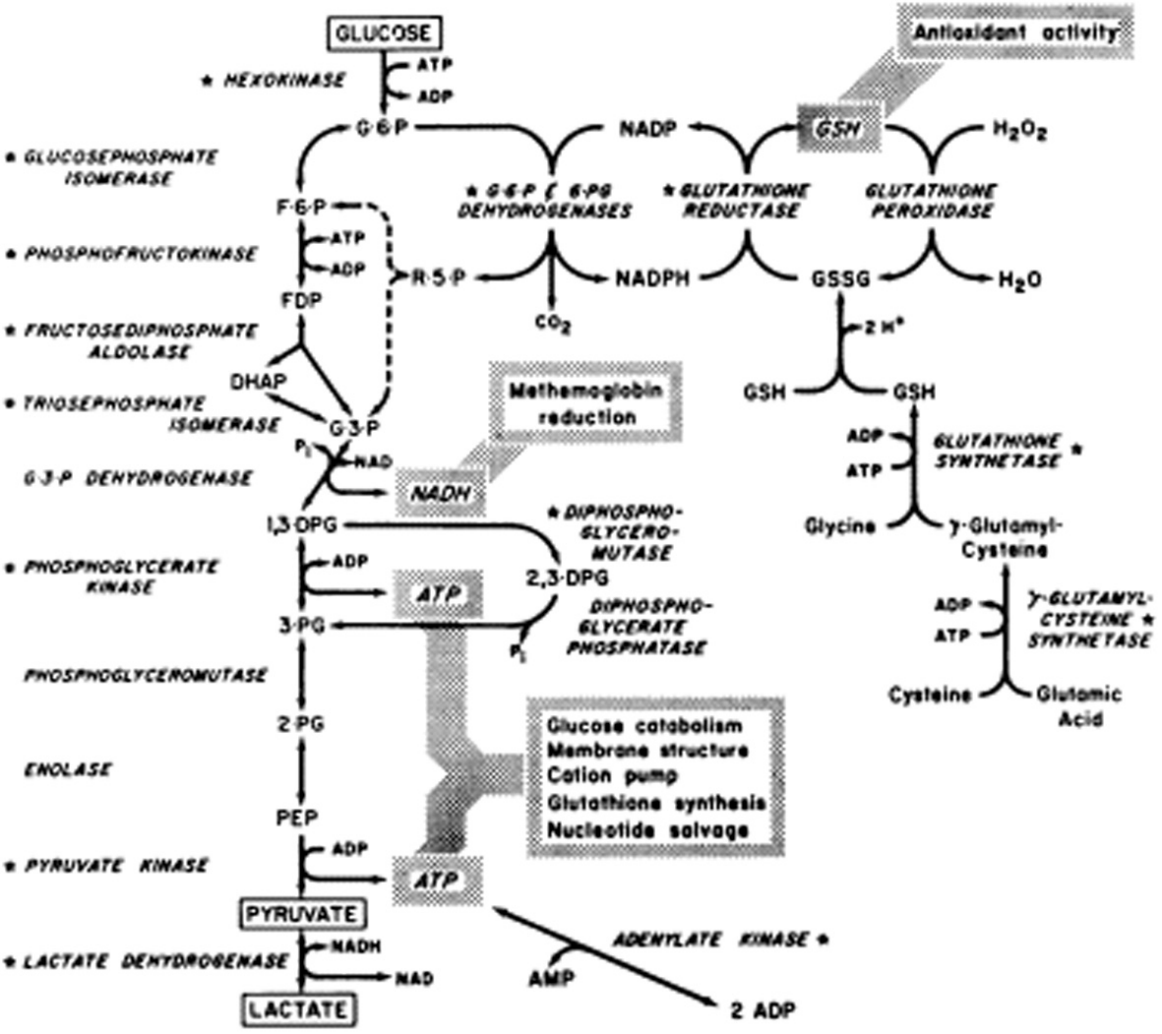
**Reprinted from Valentine (1979), with permission from Elsevier**.

As levels of the glycolytic metabolites diminish, the concentration of 6-phosphogluconate increases, as does nicotinamide adenine dinucleotide phosphate (NADPH) indicating that glycolysis is diverted down the pentose phosphate pathway (Figure 1). The pentose phosphate pathway produces NADPH which in turn reduces oxidized glutathione (GSSG), forming reduced glutathione (GSH) necessary for reduction of reactive oxygen species (ROS, Figure 1). Despite the increase of NADPH over storage there is not enough produced to maintain adequate levels of reduced glutathione; GSH falls continuously throughout the storage period and GSSG increases after day 14 (D'Alessandro et al., 2012).

### Effect on function

The metabolic changes in the stored RBC affect the function of RBCs. The build up of lactic acid and fall in pH activates the phosphatase activity of diphosphoglycerate mutase, the enzyme that dephosphorylates 2,3-DPG (Figure 1). Hence levels of 2,3-DPG decline rapidly over the first week of storage (Bennett-Guerrero et al., 2007). Molecules of 2,3-DPG modulate oxygen transport by preferentially binding to deoxyhemoglobin and thus facilitate the release of oxygen in the tissues. Loss of 2,3-DPG causes the oxygen dissociation curve of stored RBCs to shift to the left (Hamasaki and Yamamoto, 2000; Opdahl et al., 2011). Molecules of 2,3-DPG also modulate membrane stability and thus deformation properties of RBCs by interacting with band 3 (SLC4A1) and protein 4.1 (EPB41) and disrupting the link between the membrane and the cytoskeleton (Moriyama et al., 1993; Chang and Low, 2001). Binding of 2,3,-DPG to N-terminal band 3 also affects the binding of glycolytic enzymes to band 3 modulating their regulation (Rogers et al., 2013). However, 2,3-DPG is thought to be replenished post-transfusion, although this may take >24 h (Hamasaki and Yamamoto, 2000), and so the oxygen carrying ability of hemoglobin in stored RBCs recovers eventually *in vivo*. Similarly ATP is probably replenished post-transfusion when lactate can be catabolized by the liver, relieving the pH stress and restoring glycolysis. ATP can certainly be restored *in vitro* by rejuvenating with the addition of certain metabolites and warming the RBCs. However, although the metabolic parameters can be improved by rejuvenation, the remaining elements of the storage lesion are more difficult to reverse (Tchir et al., 2013).

### Oxidation

The effect of oxidative stress on RBC aging is reviewed in detail in a companion paper of this research topic “Regulation of red cell life-span, erythropoiesis, senescence and clearance” (Mohanty et al., 2014). Here we will concentrate on the effect of oxidative stress on donor RBCs in storage.

Oxidative stress damages RBCs and shortens their life span (Fibach and Rachmilewitz, 2008). Reduced glutathione (GSH) is an important anti-oxidant molecule that “mops up” ROS (Figure 1). It has been shown that the amount of GSH present in RBCs decreases after day 14 of storage, while oxidized glutathione (GSSG) increases (D'Alessandro et al., 2012). The consequence is that oxidative damage increases, and this is reflected by an increase in malondialdehyde (MDA, a marker of lipid peroxidation) and protein carbonylation. Carbonylation, a marker of protein oxidative stress, increases from day 0 to day 28 (D'Alessandro et al., 2012) and occurs mainly on membrane and cytoskeleton proteins (Kriebardis et al., 2007a; Delobel et al., 2012). Carbonylation occurs earlier and more severely in CPDA-stored than CPD-SAGM-stored RBCs probably due to increased oxidative stress in CPDA-stored RBCs (Antonelou et al., 2010). Carbonylation of RBC protein decreases after day 28 perhaps because the oxidized protein is released in vesicles (D'Alessandro et al., 2012).

Oxidative stress may also be aggravated later in storage by iron release. Hemolysis increases over storage, releasing iron, which exacerbates the situation by causing oxidative damage and further hemolysis (Collard et al., 2013). Oxidative stress may also be increased in RBCs from glucose 6-phosphate dehydrogenase (G6PD) deficient donors. These donors provide 0.3% of RBC units in New York and a high proportion of them are R_o_R_o_ phenotype (12.3% of the G6PD-deficient units in New York). This has implications for sickle cell patients; R_o_R_o_ units are used preferentially for sickle cell patients who may be adversely affected by oxidized RBCs (Francis et al., 2013).

Alterations in cytoskeletal proteins (spectrin, protein 4.1, protein 4.2, dematin, ankyrin) are a clear indicator of oxidative stress. Other indicators are the recruitment of certain proteins to the membrane, for example the stress-response protein HSP-70, which then interacts with damaged cytoskeletal proteins (Antonelou et al., 2010). Anti-oxidant enzymes such as peroxiredoxin-2 (PRDX2, previously known as calpromotin) are also recruited to the membrane during oxidative stress (Antonelou et al., 2010; D'Alessandro et al., 2012) and associate with band 3 (Matte et al., 2013). Association of PRDX2 with the membrane in sickle cells is associated with activation of the calcium-activated potassium channel (Gardos channel), dehydration and dense cell formation (Moore et al., 1997). The oxidation of hemoglobin results in the formation of hemichromes which then bind to the RBC membrane, particularly to the N-terminal domain of band 3 (Kannan et al., 1988). Oxidation of the cytoskeleton and/or band 3 is thought to disrupt the cytoskeleton (permitting greater mobility of band 3), promoting the aggregation of band 3, which can lead to antibody binding (Pantaleo et al., 2009). Indeed, association of hemichromes with band 3 causes band 3 aggregation, increased immunoglobulin attachment and erythrophagocytosis in beta-thalassemia intermedia RBCs (Cappellini et al., 1999). Oxidative damage also occurs in other membrane proteins [e.g., glyceraldehyde-3-phosphate dehydrogenase (G3PD)] inducing proteolysis, protein aggregation and cross-linking. The effects of oxidation are ameliorated when oxygen is excluded (D'Amici et al., 2007).

### Vesiculation

Vesiculation in both normal and hereditary hemolytic anemia RBCs is reviewed in detail in a companion paper of this research topic “Regulation of red cell life-span, erythropoiesis, senescence, and clearance” (Alaarg et al., 2013). Here we will concentrate on vesiculation of donor RBCs in storage.

Vesiculation during RBC storage has two important effects. Firstly the vesicles build up in the blood bag and are transfused into the patient, and secondly vesiculation causes irreversible damage to the RBCs because once membrane has been lost the RBC cannot regain its original morphology. Vesicles produced by cells can be divided into three main groups; exosomes (30–100 nm), microvesicles or ectosomes (0.1–1 μm), and apoptotic bodies (1–5 μm) (Gyorgy et al., 2011). What is less clear is the sidedness of each of these types of vesicle. This has some clinical significance because if the vesicles produced in storage are right-side out then they are likely to be maintained in the circulation for much longer. Vesiculation may be used to rid the cell of unwanted or damaged proteins/components, in which case the vesicles need to carry a signal for immediate disposal. Alternatively, the vesicles may be used to communicate between cells, in which case they need to persist in the circulation at least until they have delivered their message.

Vesicles formed in order to carry messages between cells would need to be right-side out as immediate removal by macrophages would be counter-productive. Right-side out vesicles can be formed by simple blebbing or budding of the plasma membrane (ectosomes/microvesicles) or by the production of exosomes. Exosomes are formed by initial endocytosis producing an inside-out internal vesicle which then invaginates to form a multi-vesicular body (MVB) containing right-side out exosomes. Although immature RBCs (reticulocytes) are thought to produce exosomes via formation of MVBs (Blanc and Vidal, 2010), mature RBCs lack this capacity and release only microvesicles/ectosomes (Alaarg et al., 2013). RBCs shed right-side out vesicles *in vivo* (Dumaswala and Greenwalt, 1984), and these may communicate with other intact cells. Indeed, vesicular transfer of glycosylphosphatidylinositol-linked proteins has been shown to occur, correcting the defect in paroxysmal nocturnal hemoglobinuria (PNH) RBCs (Sloand et al., 1998, 2004). These right-side out vesicles are also implicated in inflammatory and immunomodulatory responses to transfusion (Sadallah et al., 2008, 2011; Kriebardis et al., 2012).

Vesiculation may be used to remove damaged proteins from the mature RBC membrane and prolong the life of RBCs *in vivo* (Bosman et al., 2012). There is evidence that vesiculation to remove damaged protein also occurs in stored RBCs. Electron and confocal microscopy images show that during storage the morphology of RBCs changes from discocyte to echinocyte, and that vesicles appear to form at the tips of the echinocytic spicules (Tissot et al., 2010), suggesting that vesicles produced in stored RBCs are ectosomes. Ectosomes would be expected to form in a right-side out manner, which would not expose phosphatidylserine (PS; a phospholipid located exclusively on the inner leaflet of the membrane bilayer). However, studies have found that about a third of storage-induced RBC ectosomes do expose PS on their surface (Salzer et al., 2008). Ectosomes of platelet origin have also been reported to be a mixture of PS positive (20%) and PS negative (80%) vesicles (Connor et al., 2010). So the cell must have some mechanism for exposing PS at the tips of the spicules. It is possible that damaged protein destabilizes the packing of the transmembrane proteins and the cells integrity, allowing localized entry of calcium. Lipid rafts may form around the damaged protein, calcium may activate scramblases and translocation of PS to the outer leaflet and result in the formation of a PS-exposing bleb.

**Figure 2:**
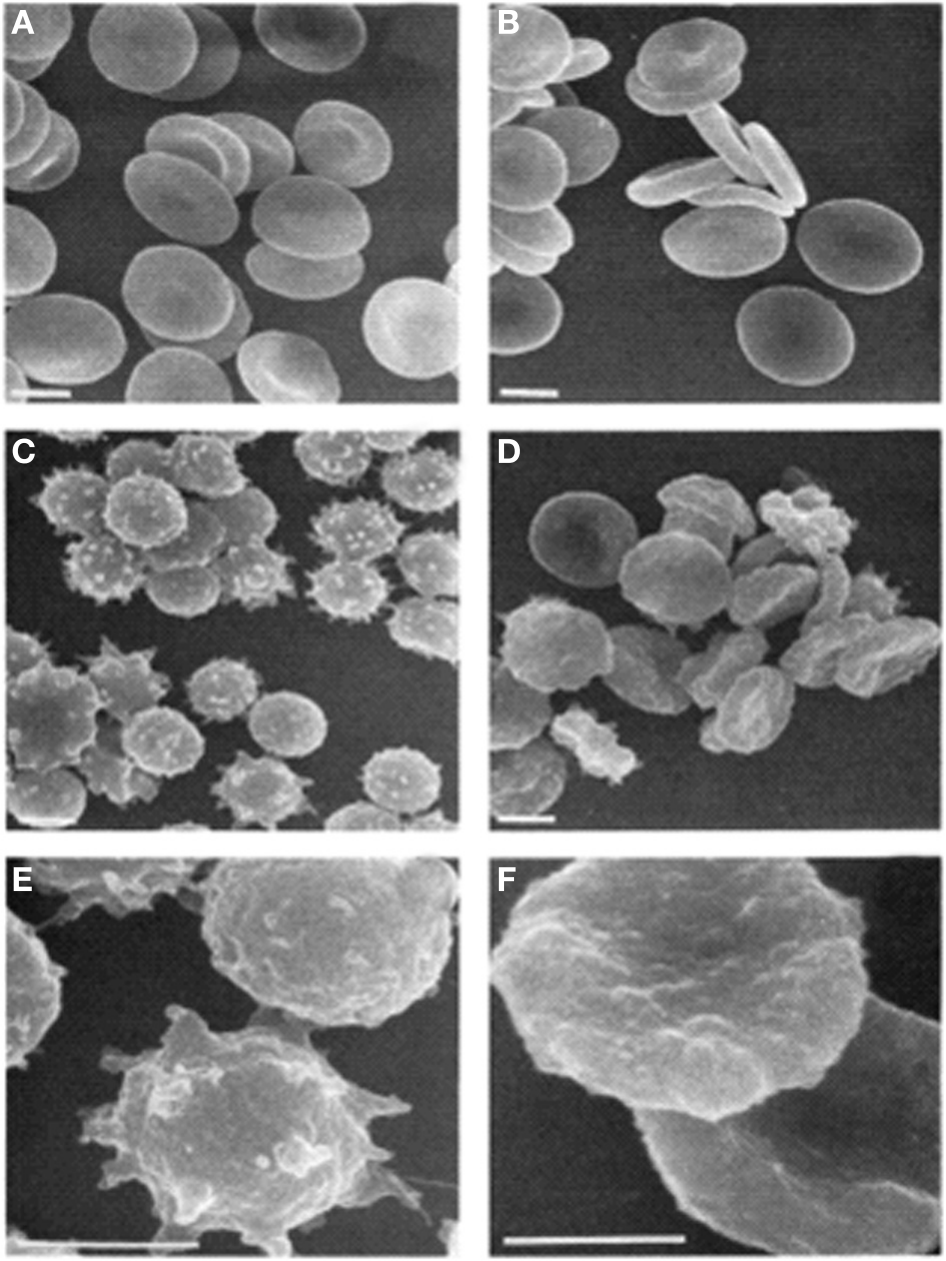
**Scanning electron micrographs of RBCs. (A)** Untreated control; **(B)** untreated Scott syndrome; **(C,E)** A23187-treated control; **(D,F)** A23187-treated Scott syndrome. Bar indicates 3 μm. Magnification in **(E,F)** is three times higher than in **(C)** through **(D)**. Cells were fixed 1 h after addition of ionophore. Reprinted from Bevers et al. (1992), with permission from Elsevier.

Indeed there is some evidence that spiculation and vesiculation may be dependent on PS exposure. In Scott syndrome, a bleeding disorder, PS exposure is inhibited in both platelets and RBCs. Loss of PS exposure in these RBCs (and platelets) impairs the formation of echinocytes and calcium-induced vesiculation (Figure 2; Bevers et al., 1992). The Scott syndrome patient was found to have a splice site mutation in *TMEM16F*, now known as anoctamin 6 (*ANO6*) (Suzuki et al., 2010). This protein has since been found to form a calcium-activated cation channel required for lipid scrambling (Yang et al., 2012).

It is worth noting that there are differences between storage-induced RBC vesicles and calcium-induced RBC vesicles, which are produced by incubating RBCs with a calcium ionophore in the presence of calcium. Both types of vesicle contain detergent-resistant material (DRM) comprising of lipid rafts and are rich in the raft proteins acetylcholinesterase, CD55, flotillins, stomatin (Salzer et al., 2008), and are free from cytoskeleton proteins (De Jong et al., 1996), although actin is present (Kriebardis et al., 2008). Storage-induced vesicles are a similar size to calcium-dependent vesicles, but contain more stomatin and less flotillin (Salzer et al., 2008). Calcium ionophore treatment was shown to produce vesicles rich in stomatin, synexin (annexin VII), and sorcin. The latter two proteins are usually cytosolic but are recruited to the membrane upon stimulation with calcium (Salzer et al., 2002). Synexin and sorcin have been reported to be recruited to the membrane in stored RBCs, leading the authors to suggest that calcium-dependent processes are likely to occur in RBCs during storage, despite the presence of citrate in the storage solution, which chelates calcium (Kriebardis et al., 2007b).

Band 3 is an important protein for the structure and morphology of the red cell because it links the membrane to the underlying spectrin cytoskeleton. This is achieved via a strong association with the linker protein ankyrin. Studies suggest that phosphorylation of band 3 disrupts its interaction with ankyrin and therefore weakens the membrane-cytoskeleton link (Ferru et al., 2011; Pantaleo et al., 2011). A high state of phosphorylation on band 3 was shown to result in echinocytic morphology and increased microvesicle release (Ferru et al., 2011), providing another mechanism for vesiculation.

Band 3 and Hb are found in vesicles throughout storage, probably representing the non-attached band 3 (Bosman et al., 2008). The amount of CD47, a marker of self, increases in vesicles during storage and this loss of CD47 from stored RBCs may affect RBC clearance post-transfusion (Anniss and Sparrow, 2002). Some proteins, for example complement receptor 1 (CR1), are enriched in vesicles (Pascual et al., 1993) but other proteins are absent from vesicles, for example aquaporin 1, suggesting that there is sorting of proteins into vesicles while others are preferentially retained (Kriebardis et al., 2008). By day 35 of storage vesicle formation proteins such as alpha SNAP can be found at the membrane (D'Alessandro et al., 2012). Vesicles also carry a large amount of immunoglobulins. Fas-related signaling molecules such as Fas-associated death domain (FADD) and caspase 8 are present in vesicles and the amount of vesicle-associated Fas and caspase 3 increases with storage time. Fas is thought to segregate to lipid raft domains, activate caspases which inhibit aminophospholipid translocase (flippase) activity and cause PS exposure (Mandal et al., 2005). As previously mentioned, about one third of vesicles expose PS (Salzer et al., 2008), and transfusion of PS-exposing vesicles may have pro-coagulatory effects leading to thrombo-embolic complications (Owens and Mackman, 2011; Rubin et al., 2012). In some situations this may be of benefit to stop bleeding (Jy et al., 2011; Kriebardis et al., 2012). PS-exposing vesicles may also recombine with other cells, labeling their surface with PS and marking them for destruction. Vesicles also carry Hb, which can increase the iron-load of the transfused patient, and can deplete nitric oxide, inhibiting vasodilation.

### Cation gradient dissipation

All RBCs (circulating or stored) have a slight permeability to monovalent cations. The RBC membrane provides a permeability barrier, enabling the cell to maintain different concentrations of ions internally, but this barrier is not perfect and ions leak down their concentration gradients. This minor leak is constantly corrected by the NaKATPase which pumps potassium into the cell in exchange for sodium and maintains a gradient such that in humans RBCs contain ~90 mM potassium and ~5 mM sodium whilst the plasma contains ~5 mM potassium and ~140 mM sodium. However, donated RBCs are stored at 4°C, and at this temperature the NaKATPase has limited functionality (Marjanovic and Willis, 1992), even before ATP becomes limiting, so cations leak across the RBC membrane unopposed until they find an equilibrium with the external medium (Wallas, 1979). As discussed above, metabolic and oxidative changes develop throughout the storage period, but the cation leak is apparent immediately and may be responsible for early changes in stored RBCs; in membrane potential, cell volume and morphology (Berezina et al., 2002). After 35 days storage the extracellular solution of a leukodepleted unit of packed RBCs contains ~40–50 mM potassium (Bawazir et al., in press). Concurrently the intracellular concentrations of monovalent cations change in stored RBC; potassium concentration is reduced and intracellular sodium levels increase, although this is rarely reported. The consensus is that cation gradients can be restored in RBCs post-transfusion. Restoration of cation gradients, after incubation with glucose at 37°C, was reported many years ago (Flynn and Maizels, 1949) however this study used 6-day stored RBCs, when ATP levels would be high. Conversely, a more recent study showed that overnight incubation at 37°C of 35-day stored RBCs increased potassium leakage, hemolysis, PS exposure, and vesiculation (Burger et al., 2013).

**Figure 3:**
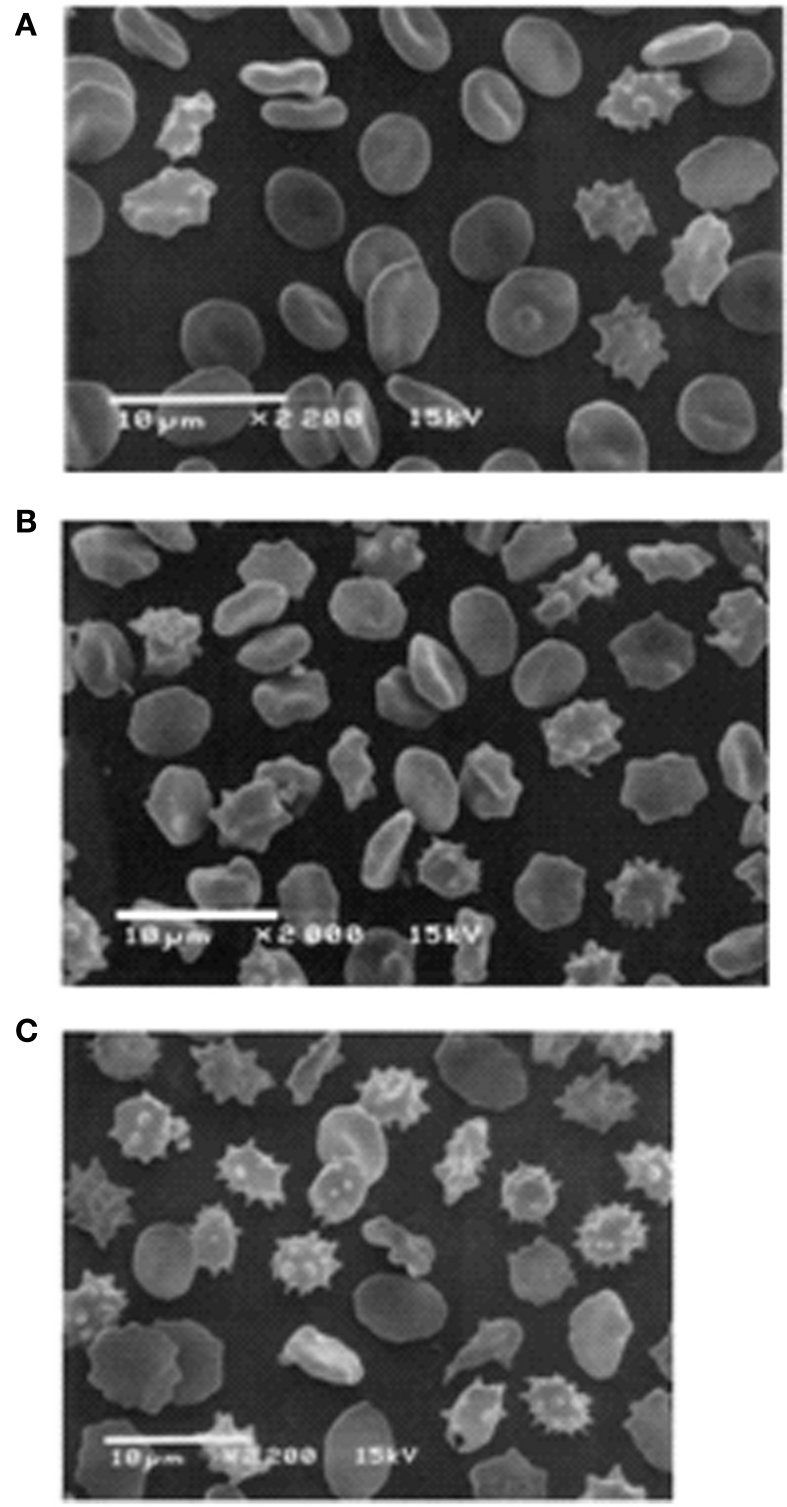
**Scanning electron microscope pictures of RBC**. Stored RBC on the 5th **(A)**, 14th **(B)**, and 42nd **(C)** day of storage. **(A)** Discocytes dominate among the cell population, and only a few irreversibly changed RBC can be seen. **(B)** Numerous echinocytes and spheroechinocytes can be seen. **(C)** Spheroechinocytes and degenerated forms dominate among irreversibly changed cells. Cells stored in adenine saline solution (AS-3 contains saline, adenine, glucose, phosphate, and citrate). Reprinted from Berezina et al. (2002), with permission from Elsevier.

The altered cation gradient across the RBC membrane of RBCs stored in SAG-M has a distinct effect on cell volume and shape. This can be seen by comparing circulating RBCs with stored RBCs. Both types of cell develop oxidized protein and lipid and vesiculate, however cell volume changes differ in the two cell types. In circulating RBCs the cells start off large and become small, in stored RBCs the MCV becomes larger throughout storage. This difference may in part be due to the presence or absence of calcium. The dehydration of old circulating RBCs is thought to involve the calcium-activated Gardos channel whereas the storage solution of stored RBCs contains some citrate, which chelates calcium. The different volume changes in circulating and stored RBCs suggests the mechanism of vesiculation may be different in the two cells types. In circulating RBCs the cation gradient is maintained and the shape, volume and cytoskeleton integrity are maintained until the cell becomes damaged. Then controlled vesiculation occurs in order to remove damaged protein (Bosman et al., 2012). About 20% of the RBC membrane may be lost in this way during the 120 day lifetime of a RBC. Eventually small, dense, spherocytes result and are removed from the circulation by phagocytosis. In stored RBCs the cation gradient is lost gradually and the cell shape and volume are altered. The cells swell, weakening the cytoskeleton which is also weakened by oxidation and ATP loss. The membrane becomes unstable, echinocytes form and membrane is lost by vesiculation, seemingly a much less controlled mechanism (Figure 3). Even at day 5 of storage the membrane appears less tightly controlled with some swollen, misshapen discocytes present (Figure 3A).

By day 21 of storage the osmotic fragility of the RBCs is increased and more than 50% of the cells display non-discocyte morphology (Blasi et al., 2012). By day 35 of storage the morphology of about 25% of RBCs is irreversibly altered; the cells have lost membrane through vesiculation and become spherocytic (spheroechinocytes, spherostomatocytes, spherocytes) (Blasi et al., 2012). About 25% of long-stored RBCs are immediately removed from the circulation post-transfusion (Luten et al., 2008). It is likely that the irreversibly-altered, echinocytic spherocytes in long-stored RBCs form this fast-removed population (Beutler et al., 1982). It may not simply be PS expression that triggers their removal, indeed PS exposure on stored RBCs remains quite low (Verhoeven et al., 2006), only beginning to increase around day 28 of storage (Dinkla et al., 2013). It may also be due to the rigidity of these cells, which would resemble that of hereditary spherocytosis cells.

## The order of the storage lesion

Many studies have tried to unravel the order in which the different elements of the storage lesion occur. The current accepted theory holds that during storage ATP levels fall, ROS increases and the RBC membrane becomes oxidized. This causes disruption of the cytoskeleton, aggregation of band 3 and release of vesicles. There is significant evidence supporting this course of events. ATP levels, after rising during the first week of storage, fall away from day 7 onwards (D'Alessandro et al., 2012). ROS levels increase gradually for the first week of storage and then rapidly increase to a maximum by the second week of storage (D'Alessandro et al., 2012). Oxidation is shown to reduce the spectrin–actin interactions during storage (Wolfe et al., 1986) and correlates with vesicle release (Wagner et al., 1987). Aggregation of the mobile pool of band 3 coincides with increased ROS and oxidation of the RBCs (Kriebardis et al., 2007a; Karon et al., 2012; Arashiki et al., 2013) and occurs before vesiculation (Karon et al., 2009). Deformability of stored RBCs decreases throughout storage as cells lose membrane and become spherical (Bennett-Guerrero et al., 2007). Consequently much effort has been put into maintaining ATP levels in stored RBCs.

### Association of MCV and RDW with the cation leak

However, this sequence of events ignores the impact of the cation leak which occurs from day 1 of storage. It is possible that the change in cation distribution initiates some of the other components of the storage lesion. The cation leak in stored RBCs causes a redistribution of monovalent cations but also an overall uptake of base (and therefore water) which causes the cells to swell (Flynn and Maizels, 1949). In circulating RBCs loss of cation gradients and cell swelling may activate the Gardos channel, resulting in loss of potassium (and cell shrinkage), but this mechanism requires calcium which is reduced in stored RBCs due to the presence of citrate in the CPD-SAGM. So the overall effect of cation changes is for there to be an increase in mean cell volume (MCV). Indeed the MCV of RBCs stored in SAG-M increases steadily from day 1 and throughout storage (Antonelou et al., 2012; Bawazir et al., in press). However, during storage some cells lose membrane by vesiculation and become smaller and spherocytic. Therefore, the remaining cells must be even more swollen for the MCV to continue to rise, and this is reflected in the steady increase in red cell distribution width. Figure 3 shows the variation in size and shape of red cells stored in AS-3. By day 35 of storage (Figure 3C) the cells range from large and misshapen to small, spherocytic echinocytes. Even at day 5 of storage (Figure 3A) both these cell types can be seen and this is before any ATP loss or a significant rise in ROS. ATP levels are known to rise over the first 7 days of storage, and ROS and protein carbonylation increase only slowly for the first 7 days, then rapidly between day 7 and day 21, when they reach a plateau (D'Alessandro et al., 2012; Suppl Figure S2). So, although ROS may contribute to these early morphological changes in the RBCs, changes in cation gradients probably play an important role.

It should be noted here that this increase in MCV does not always occur when RBCs are stored in other storage media. Although all stored RBCs leak cations, regardless of the storage medium, different storage media affect MCV and osmotic fragility in different ways (Zehnder et al., 2008; Veale et al., 2011). The MCV of RBCs stored in PAGGSM remains fairly constant throughout storage and the MCV of RBCs stored in Erythrosol-4 decreases throughout storage (Veale et al., 2011). These storage media related differences were explained by differences in vesiculation (Veale et al., 2011), although another study found no difference in vesiculation (Zehnder et al., 2008) and vesiculation can vary enormously between different donors (Rubin et al., 2008; Lion et al., 2010). These storage media related differences cannot be explained by differences in Gardos channel activity. Although the MCV of RBCs stored in Erythrosol-4 decreased steadily through storage, Erythrosol-4 contains 25 mM Na-citrate (Veale et al., 2011).

### Association of PS exposure with the cation leak

The impairment of PS exposure in Scott syndrome, discussed above, suggests PS exposure may be a prerequisite for the formation of echinocytes and RBC vesiculation. It has been shown that the high intracellular potassium concentration of fresh RBCs inhibits lipid scrambling activity (Wolfs et al., 2009). So it follows that when the stored RBC leaks cations and the intracellular potassium levels go down, scrambling may increase and more PS may be exposed. At the same time the cation leak causes overhydration of the stored RBCs in SAG-M (as discussed above) resulting in large misshapen cells. Together these two effects of the cation leak, overhydration and PS exposure, may initiate the early changes in RBC morphology creating protrusions or spicules on the cell membrane. One study appears to support this hypothesis. The authors found that reduced intracellular potassium caused decreased flippase activity, causing PS exposure and vesiculation (Burger et al., 2013). This was an *in vitro* study that used an overnight incubation at 37°C to mimic post-transfusion conditions. It showed that flippase activity was reduced under these conditions, however the study did not report the ATP levels of the stored RBCs and lack of ATP may have contributed to these findings. In another study it was reported that scramblase activity is virtually absent during RBC storage, and flippase activity, although reduced after 21 days storage due to lack of ATP, can be restored if metabolic changes are corrected (Verhoeven et al., 2006).

### Association of vesiculation with the cation leak

Vesiculation increases with prolonged storage but increases markedly after day 21 (Rubin et al., 2008; Lion et al., 2010). Vesiculation seems to occur partly as a result of the cation leak causing cell swelling and PS exposure (as discussed above), and partly by the oxidation of the membrane in particular the cytoskeleton. Certainly oxidation of cytoskeleton/spectrin has been shown to lead to vesiculation. Prolonged storage weakens the spectrin–actin-protein 4.1 interactions and this effect is probably due to oxidation of spectrin or other cytoskeletal proteins and can be reversed in part by treatment with a reducing agent, dithiothreitol (Wolfe et al., 1986). It has also been shown that there is variation in the number of vesicles produced from RBCs stored in different storage media; those media that efficiently combat oxidative stress produce fewer vesicles (Dumaswala et al., 1996; Antonelou et al., 2010; Veale et al., 2011). Similarly, another study has shown that the expression of aging markers such as aggregation and proteolysis of band 3 and increased binding of hemoglobin and autologous antibodies are more pronounced in CPDA than CPD-SAGM suggesting that oxidation is involved (Antonelou et al., 2010). So although the cation leak may initiate changes in the membrane that lead to vesiculation in the early stages of storage, oxidation of the membrane doubtless plays a significant role in vesiculation after the second week of storage.

### Association of oxidation with the cation leak

Interestingly, oxidation of stored RBCs and the effects of the cation leak in stored RBCs appear to be intertwined. Some red cell concentrate units are subjected to doses of gamma irradiation in order to prevent graft-vs.-host disease in vulnerable patient groups. This process is associated with increased oxidative damage to the red cell membrane as well as an increased potassium leak (Serrano et al., 2013). It has been shown that oxidation of the cytoskeleton may exacerbate the cation leak by weakening the integrity of the membrane (Deuticke et al., 1984; Ney et al., 1990). Lipids are also oxidized and lipid hydroperoxides may permit a deformation-dependent leak of monovalent cation from erythrocytes (Sugihara et al., 1991) although this is disputed by others (Deuticke et al., 1987). Indeed the oxidation and deformation effects on the RBC cation leak are synergistic (Ney et al., 1990). Equally, the cation leak may also exacerbate oxidation of the stored RBCs. Long-term storage of human RBCs is associated with a decrease in the concentration of glutathione because of a reduced rate of synthesis (Whillier et al., 2011). This was found to be caused by both decreased ATP concentration and reduced amino acid transport (Whillier et al., 2011). As certain amino acid transporters are sodium dependent, such as SLC1A5 the Na-dependent neutral amino acid transporter that transports glutamine and asparagine, the reduction in amino acid transport could in part result from loss of sodium gradient across the membrane.

The above discussion by no means resolves the order in which the different elements of the storage lesion occur but may go some way to highlight how all of these factors are interlinked. Hopefully it also makes a case for more attention to be paid to the cation leak and its effect on the other components of the storage lesion. Reduction of the cation leak, perhaps by improvements in storage media, may delay or ameliorate other components of the lesion. Something that has become clear recently is that there is wide variation in severity of the storage lesion amongst blood donors, in particular variation in cation leak.

## Variations in donor red cell properties

Individual blood donors can show a wide variation in the properties of their red cells. This can be as a result of lifestyle and diet. Indeed, it has already been discussed in this series of articles that some athletes' circulating red cells represent a younger population because of intravascular hemolysis during impact sports (Mairbäurl, 2013). At the other end of the spectrum, it is well established that an increase in erythrocyte MCV often accompanies chronic alcoholism (Wu et al., 1974). It has also been observed that there is a large variation in the number of RBC vesicles shed by different donors, but the reason for this is unknown (Rubin et al., 2008; Lion et al., 2010). Some blood parameters vary according to age and gender; but in addition to this, variation in donated cells can have a genetic basis. There is evidence from genome-wide association studies that single nucleotide polymorphisms (SNPs) in certain genes are linked to variations in the properties of donors' red cells, such as MCV and mean cell hemoglobin (MCH) among others (Ganesh et al., 2009). Indeed, approximately 25 percent of black blood donors have elevated RBC sodium (Na_i_) level compared with white donors, probably due to an unknown genetic change. This elevation results in a significant increase in the mean Na_i_ from black (9.00 ± 2.96 mmoles/L RBC) as compared to white blood donors (7.04 ± 1.48 mmoles/L RBC, *p* < 0.001) (Wallas et al., 1982).

**Figure 4:**
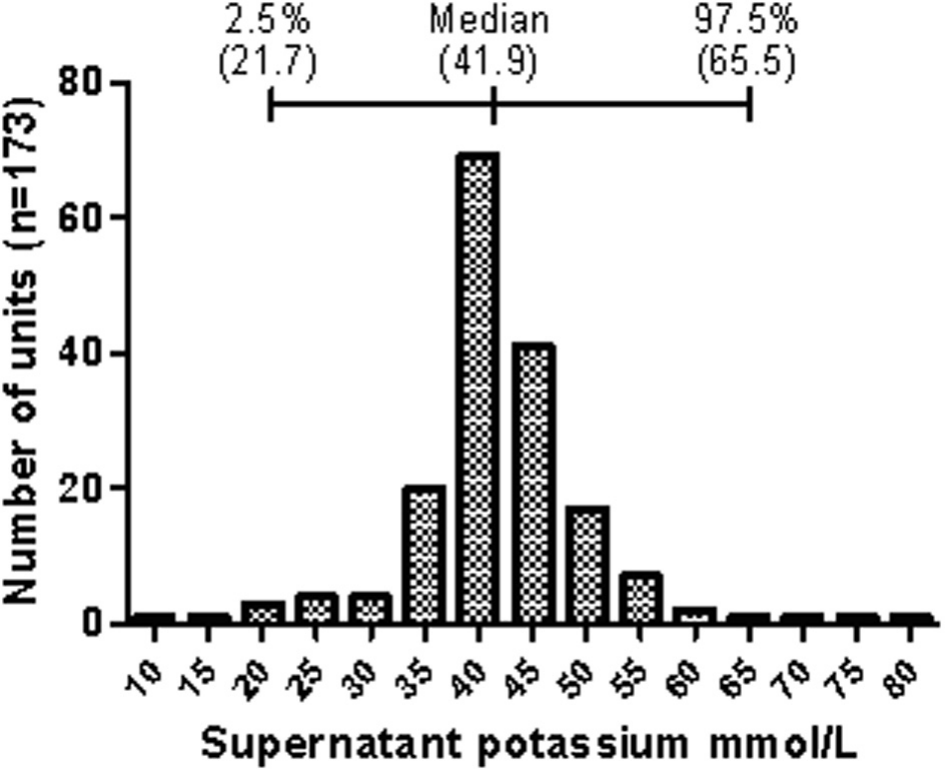
**Distribution of supernatant potassium concentration of stored donor red cell units**. The graph shows the distribution of the potassium concentration in the supernatant of red cell units at day 35 of refrigerated storage as collected by the Components Development Laboratory, NHSBT. Data represents randomly selected units collected between 2003 and 2006. The median and median percentile data are shown above the bar chart. Reprinted from Bawazir et al. (in press), with permission.

We have shown that in the donor population there is a natural variation in the rate of the cation leak across the red cell membrane (Bawazir et al., in press). In everyday life this variation is of no consequence, because the correcting action of the NaKATPase easily maintains the cation gradients necessary for cellular volume control and hydration. Once the red cells are donated and stored, this natural variation becomes measureable as the action of the NaKATPase is ablated by cold-storage. At day 35 of storage most units have 40–45 mM extracellular potassium but some units have much higher levels (up to 80 mM), and some have much lower levels (10 mM) (Figure 4). The majority of variation in potassium levels in units after 35 days' storage is probably due to environmental factors, but there may be genetic markers that are associated with a particularly high or low rate leak.

### FP-Cardiff and the high-rate cation leak

Consistent with the idea that some aspects of the red cell cation leak can have a genetic basis, we have identified a SNP in a red cell membrane protein that co-segregates with a high cation leak condition in a three-generation family. This mutation has so far been found in two unrelated UK blood donors (Bawazir et al., in press). This particular condition, called FP-Cardiff, forms part of a larger family of disorders that share the common feature of cation-leaky red cells. Collectively, they are known as the hereditary stomatocytoses (Stewart, 2004; Gallagher, 2013). Unlike the other members of the hereditary stomatocytoses FP-Cardiff has relevance to blood donation and storage because carriers of this mutation are not anemic, show no disease symptoms and therefore may become blood donors. However, once their blood is cooled for storage the red cell membrane exhibits an unusually high rate of cation leak such that potassium levels in the unit can be 40 mM by day 5 (Bawazir et al., in press). Ultimately, as with normal units, the levels of supernatant potassium in FP-Cardiff units will plateau once an equilibrium is reached between the intracellular and extracellular space.

Under normal circumstances the transfusion of high-potassium units is unlikely to affect the recipient adversely, because the transfusion of blood is slow and the relatively large volume of the recipient's own circulating blood will dilute the potassium to safe levels. However, in certain situations, such as large volume or exchange transfusions for neonates, there is a requirement for low-potassium units. In these cases it is normal practice to use blood that has been stored for less than 5 days in order to avoid high supernatant potassium. Unusually high levels of potassium may be present in short-stored blood packs if they have come from a donor with FP-Cardiff (or other condition that results in a high rate of potassium leak during blood storage). The consequences of a high potassium transfusion can be serious, including cardiac arrest (Vraets et al., 2011). Interrogation of the available databases suggests that FP-Cardiff could be present in 1 in 500 people in the European population (Bawazir et al., in press).

Aside from the risk of transfusion-associated hyperkalemia, it is not yet known if the accelerated loss of the cation gradients in high-leak red cells speeds up or exacerbates the other features of the storage lesion. As the correct hydration of red cells is so dependent on these cation gradients one might expect that the changes in MCV and red cell morphology that are observed over storage may be accelerated in FP-Cardiff units. An increase in the formation of microvesicles and/or sphero-echinocytic cells would be a concern because of the potential inflammatory and thrombotic effects of microvesicles, and the fact that the spleen will immediately remove the majority of spherocytic cells (Safeukui et al., 2012). Further work to characterize FP-Cardiff red cells and establish how they respond to prolonged storage is currently ongoing in our laboratory. The study of these cells will be a useful tool to investigate the relationship of the cation leak with vesiculation, membrane oxidation and other hallmarks of the storage lesion.

## Concluding remarks

There is still some debate among clinicians over whether the duration of blood storage really matters (Cheuk, 2012; Wang et al., 2012). It is, however, undeniable that certain changes do occur once the blood donation is given, the pack is processed and the cells are kept in cold storage for as long as 42 days. A large amount of work has been done to characterize the red cell storage lesion, and we are now beginning to understand the order in which the various elements occur during storage. One of the problems associated with older blood is the loss of cation gradients across the red cell membrane. This aspect has hitherto not received the degree of attention afforded to other lesion hallmarks such as vesiculation and oxidative damage. It appears to be the case that most elements of the lesion, if not all of them, show some degree of inter-dependence. As such, the contribution of the cation leak is beginning to be better understood but more work will be necessary to untangle the intricacies of these complex relationships. Further studies to understand the cause and effect of the different elements of the lesion are key to ameliorating the initiating factor(s) and reducing the lesion.

## Author contributions

All authors contributed to the manuscript preparation.

### Conflict of interest statement

The authors declare that the research was conducted in the absence of any commercial or financial relationships that could be construed as a potential conflict of interest.
